# Retained tracheostomy stay suture with migration into the glottic airway: A case report

**DOI:** 10.1002/ccr3.6625

**Published:** 2022-11-20

**Authors:** J. Dixon Johns, Andy M. Habib, Ronak B. Dixit

**Affiliations:** ^1^ Department of Otolaryngology‐Head and Neck Surgery MedStar Georgetown University Hospital Washington DC USA; ^2^ Georgetown University School of Medicine Washington DC USA; ^3^ Department of Otolaryngology‐Head and Neck Surgery MedStar Washington Hospital Center Washington DC USA

**Keywords:** decannulation, stay suture, trachea, tracheotomy

## Abstract

The stay‐suture technique (SST) helps ensure safe replacement of the tracheostomy tube after accidental decannulation. We describe a patient found to have a retained stay suture in the glottis 2 weeks post‐decannulation. It is important to appreciate the possible complications associated with SST, including airway compromise, infection, and laryngospasm.

## Graphical abstract

We review the literature and present a patient case wherein the tracheostomy stay suture migrated to the airway. Although no significant complications arose, airway foreign bodies present great risk for airway compromise, infection, or laryngospasm.

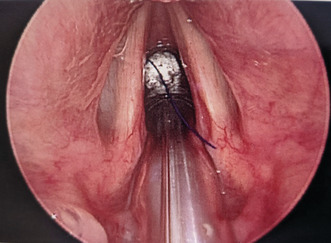

## 1 INTRODUCTION

Tracheostomy is a potentially life‐saving procedure to secure a patient's airway. There have been many techniques developed in order to mitigate risks of tracheostomy tube displacement, including the stay‐suture technique (SST) and Bjork flaps, among others. SST refers to the placement of sutures either around a tracheal ring or between the skin and lower trachea to ensure correct replacement of the tube into trachea in case of accidental decannulation and to reduce risk of false passage. While SST has been shown to reduce mortality associated with accidental decannulation, it also presents potential complications associated with introducing foreign material in close proximity to the airway. This report reviews the current literature regarding retained tracheostomy stay sutures and highlights a unique presentation of a patient who presented with a retained tracheostomy stay suture in the glottic airway following decannulation. The MedStar Health Institutional Review Board determines all case reports to be exempt from review.

## 2 CASE REPORT

A 53‐year‐old male patient presented to the emergency department with left‐sided weakness of extremities, left‐sided facial droop, and altered mental status. The patient was intubated in the emergency department due to progressive lethargy, and subsequent imaging showed a right thalamic hemorrhagic stroke. The patient was extubated the following day, but required re‐intubation in the intensive care unit (ICU) within 2 days following hypoxic respiratory failure from suspected aspiration pneumonia. The patient remained intubated for 12 days and continued to fail spontaneous breathing trials due to altered mental status. At this time, the patient was recommended for open tracheostomy and gastrostomy tube placement with general surgery, which was performed without complication. During the tracheostomy operation, a 7.0 Shiley cuffed tracheostomy tube was placed with three Prolene stay sutures (one suture superiorly on a trapdoor incision of the trachea, and two lateral sutures through the trachea along the left and right aspects of the tracheotomy, respectively). On postoperative Day 7, the tracheostomy tube was changed to a 6.0 Shiley cuffed tracheostomy tube. The stay sutures remained in place. Approximately 3 weeks after surgery, the patient was weaned from the ventilator and ultimately discharged from the hospital to a rehabilitation facility with the tracheostomy tube in place. The patient continued outpatient swallow rehabilitation by the Speech and Language Pathology (SLP) team with primary nutrition via gastrostomy tube due to concern for severe swallow deconditioning with concern for pharyngeal dysphagia. He was decannulated at the facility 6 weeks after tracheostomy tube placement without complication, although it is unclear if it was an intentional or accidental decannulation. He was clinically stable without the tracheostomy, and the facility opted not to replace the tube, allowing the stoma to close by secondary intention. He reported subjective hoarseness, but denied any shortness of breath.

A few weeks later, fiberoptic endoscopic evaluation of swallowing (FEES) was performed by the SLP team at the rehabilitation facility to evaluate swallow function. During flexible endoscopy, there was a string‐like object noted in his airway on examination, and the patient was sent to the emergency department for further evaluation. At the hospital, the Otolaryngology team performed flexible laryngoscopy in the emergency department, which confirmed the presence of what appeared to be a Prolene suture at the level of the glottis without visualization of whether the suture remained anchored to soft tissue. The patient's stoma had closed with minimal granulation and no evidence of suture externally. The patient remained clinically stable from an airway perspective, and there was a discussion whether to attempt removal with awake flexible bronchoscopy versus operative direct laryngoscopy under general anesthesia. Because it could not be determined whether the suture remained anchored to the trachea and the potential risk of laryngospasm with manipulation, the decision was made to proceed with operative direct laryngoscopy for further evaluation. After obtaining consent, the patient was urgently taken to the operating room for direct laryngoscopy and removal of the foreign body.

**FIGURE 1:**
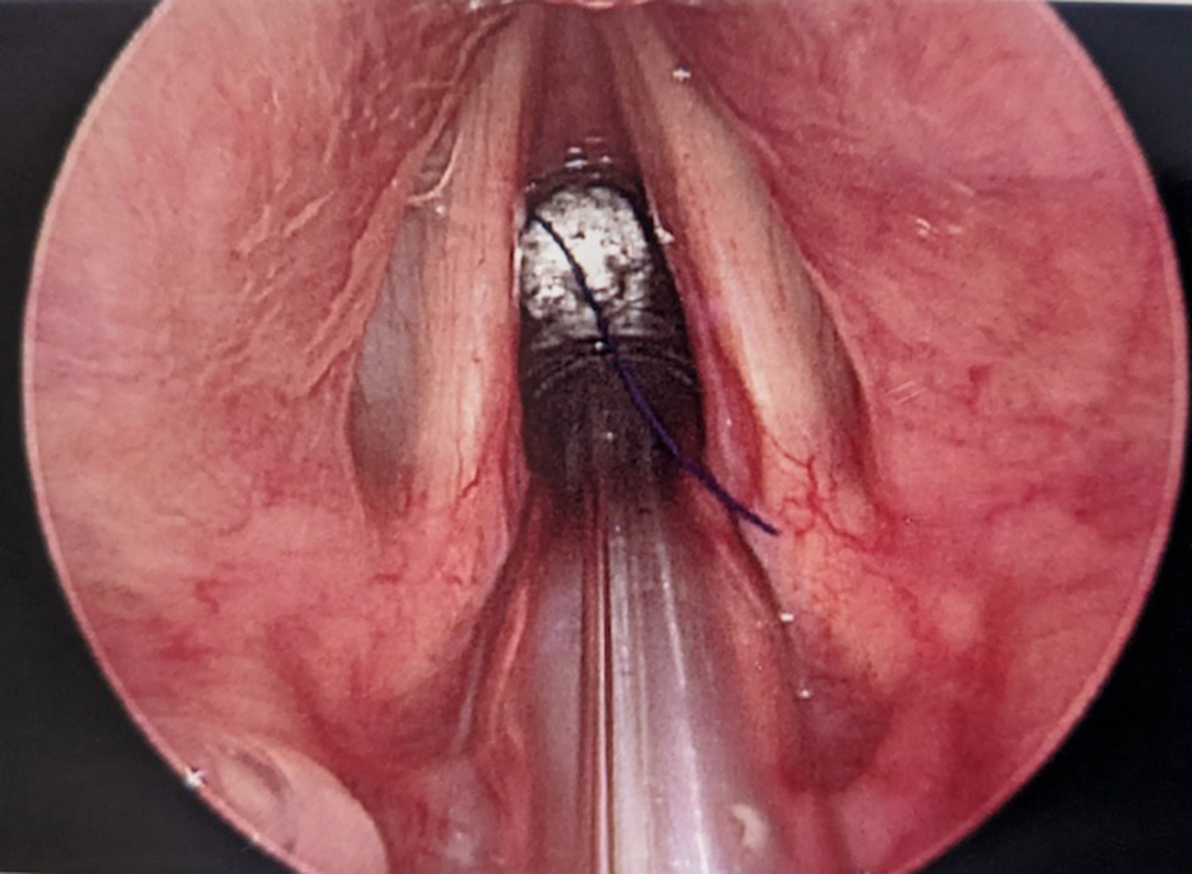
Intraoperative endoscopic visualization of a retained stay suture with migration into the airway at the level of the glottis.

The patient was orotracheally intubated uneventfully by the anesthesia team under direct visualization with video‐assisted laryngoscopy with 6.0 endotracheal tube with care to avoid the foreign body during intubation. Next, direct laryngoscopy was performed using a Lindholm laryngoscope and then placed into suspension. The airway was evaluated with use of rigid 0‐degree Hopkins rod telescope. Upon inspection of the glottis, a Prolene suture was noted lodged between the endotracheal tube cuff and the glottic airway (Figure 1). Using Ossoff‐Karlan grasping forceps, the suture was palpated and noted to be free of the soft tissue. The suture material was then grasped and easily removed without evidence of trauma or bleeding. The cuff of the endotracheal tube was then deflated and the tube carefully withdrawn for further inspection of the trachea and bilateral mainstem bronchi with rigid endoscopy. Minimal granulation tissue was noted at the level of the first and second tracheal ring at the site or prior tracheostomy. No further foreign body was identified. The endotracheal tube was replaced, and the patient was turned back over to the anesthesia team for extubation. The patient uneventfully recovered with no complication.

## 3 DISCUSSION

This case reports a patient with a retained tracheostomy stay suture that migrated into the glottic airway. The retained foreign body resulted in an interventional procedure requiring intubation under general anesthesia and admission to the hospital for observation. Although there were no catastrophic complications associated with this patient presentation, airway foreign bodies present a risk for airway compromise, nidus for infection, or laryngospasm. Retained foreign bodies, especially in close proximity to the airway, are an example of a surgical “never event.” Although stay sutures are intentionally left in place following tracheostomy, it is of utmost importance to ensure careful removal when the stoma has matured and the sutures are no longer indicated. While an uncommon complication in the literature, this report highlights importance of accounting for all foreign materials during management of the airway even in the weeks after the procedure is completed.

Accidental tracheostomy tube decannulation presents a life‐threatening complication, with incidence rate reported between 0.35% and 2.7%, with tracheostomy complication mortality rates ranging from 0.5% to 3%, due primarily to accidental decannulation and tube obstruction.^1^ SST represents a procedure to reduce the risk of mortality associated with accidental decannulation. One study compared SST vs. traditional tracheostomy without SST, finding that SST (*n* = 104) experienced no deaths while traditional tracheostomy had three deaths due to unexpected decannulation (*n* = 101, *p* = 0.024).^1^ While SST may reduce adverse events due to accidental decannulation, the risks of this technique must be taken into consideration including presumed increased operative time to place the stay sutures as well as the risk of retained foreign body.

The importance of the removal of tracheostomy sutures, including stay sutures, is well established in the tracheostomy literature. Although there is no definite recommendation on the use of sutures during routine tracheostomy for prolonged ventilation, the Clinical Consensus Statement (CCS) regarding tracheostomy tube care emphasizes the importance of the removal of any non‐absorbable suture material from the surgical site prior to discharge from the hospital, usually during the first tracheostomy tube exchange.^2^ This recommendation provides a protocol for ensuring that there is no retained suture removal that may prevent in infection, inflammation, or migration, as seen in this case report. Furthermore, evidence of suture material migration has been well established in the literature. Although there is no definitive mechanism for this phenomenon, there have been proposed theories including movement of the material through soft tissues due to the body's immune reaction to a foreign body or the contractile forces during wound healing.^3^

There are only three other case reports in the literature describing migration of a tracheostomy stay suture into the airway. Rachakonda et al.^4^ describe a patient who required surgical tracheostomy placement following vehicular trauma and subsequently downsized to a fenestrated tracheostomy tube. In the week following tube exchange, patient experienced increased secretions, tachycardia, hypertension, and hypoxia, with nursing staff noting string‐like material extruding from tracheostomy tube. Upon examination of tracheostomy tube during partial removal, it was noted that the stay suture had migrated into the stoma through the fenestration in the tracheostomy tube. Another report by Joshi et al.^5^ describes a patient who had a history of tracheostomy tube placement following hypercapnic respiratory failure with subsequent successful decannulation who had incidental anterior tracheal mucosal irregularity on routine chest imaging for lung transplant evaluation. Flexible bronchoscopy revealed retained suture material at the previous tracheostomy site from retained stay suture. Brown et al.^6^ describe a patient complaining of throat irritation and cough 9 years following decannulation of a tracheostomy tube. In office, flexible laryngoscopy revealed a suture extending from tracheal wall through the glottis. While in all cases the suture material was removed without complication, each case, including our own, required an extra procedure under general anesthesia, which could have been prevented with proper postoperative care.

Although the SST represents a potentially life‐saving intervention, this report highlights the importance of careful attention to remove the stay sutures when no longer necessary. This may be accomplished by the development of a post‐tracheostomy protocol to ensure removal of the stay sutures when the tracheostomy tube is downsized, during first tracheostomy tube exchange, or before the patient is discharged from the facility.

## 4 CONCLUSION

Stay‐suture technique is useful in preventing complications from accidental decannulation, but as with all techniques, there can be drawbacks. Here, we report an additional case of a preventable complication related to SST with migration of foreign body into the glottic airway. Although a retained stay suture migrating into the airway rare, we feel that bringing attention to this “never event” is important as tracheostomy technique continues to evolve. One strategy to prevent this would be to develop a protocol per the CCS guidelines on tracheostomy care to remove the stay sutures once the patient is deemed appropriate for tracheostomy tube exchange or prior to discharge from facility.

## AUTHOR CONTRIBUTIONS

**Andy M. Habib:** Writing – review and editing. **Ronak B. Dixit:** Conceptualization; formal analysis; investigation; writing – review and editing. **J. Dixon Johns:** Conceptualization; formal analysis; writing – original draft; writing – review and editing.

## CONFLICT OF INTEREST

None.

## CONSENT

Written informed consent was previously obtained from the patient to publish this report in accordance with the journal's patient consent policy.

## Data Availability

Data available on request due to privacy/ethical restrictions.

## References

[1] Lee SH , Kim KH , Woo SH . The usefulness of the stay suture technique in tracheostomy. Laryngoscope. 2015;125(6):1356‐1359. doi:10.1002/lary.25083 25512174

[2] Mitchell RB , Hussey HM , Setzen G , et al. Clinical consensus statement: tracheostomy care. Otolaryngol Head Neck Surg. 2013;148(1):6‐20. doi:10.1177/0194599812460376 22990518

[3] Ryu KJ , Ahn KH , Hong SC . Spontaneous complete migration of suture material after subcuticular continuous suture in cesarean section: a case report. BMC Surg. 2014;14:103. doi:10.1186/1471-2482-14-103 25481274 PMC4268889

[4] Rachakonda KS , Vangikar MM , Simmons EG . An intermittent foreign body in the airway: a case report. Crit Care Resusc. 2001;3(3):173‐175. 16573499

[5] Joshi M , Budev M , Machuzak M . Retained sutures in the trachea. J Bronchology Interv Pulmonol. 2010;17(3):236‐237. doi:10.1097/LBR.0b013e3181e64bcc 23168890

[6] Brown DJ , Pitman MJ . Retained tracheotomy suture: nine years of morbidity. Otolaryngol Head Neck Surg. 2010;142(4):626‐627. doi:10.1016/j.otohns.2009.11.022 20304292

